# Endovascular Treatment of Venous Sinus Stenosis in Idiopathic Intracranial Hypertension: Complications, Neurological Outcomes, and Radiographic Results

**DOI:** 10.1155/2015/140408

**Published:** 2015-06-04

**Authors:** Robert M. Starke, Tony Wang, Dale Ding, Christopher R. Durst, R. Webster Crowley, Nohra Chalouhi, David M. Hasan, Aaron S. Dumont, Pascal Jabbour, Kenneth C. Liu

**Affiliations:** ^1^Department of Neurological Surgery, University of Virginia, Charlottesville, VA 22908, USA; ^2^Department of Radiology, University of Virginia, Charlottesville, VA 22908, USA; ^3^Department of Neurosurgery, Thomas Jefferson University, Philadelphia, PA 19107, USA; ^4^Department of Neurological Surgery, University of Iowa, Iowa City, IA 52242, USA; ^5^Department of Neurological Surgery, Tulane University, New Orleans, LA 70112, USA

## Abstract

*Introduction.* Idiopathic intracranial hypertension (IIH) may result in a chronic debilitating disease. Dural venous sinus stenosis with a physiologic venous pressure gradient has been identified as a potential etiology in a number of IIH patients. Intracranial venous stenting has emerged as a potential treatment alternative. *Methods*. A systematic review was carried out to identify studies employing venous stenting for IIH. *Results*. From 2002 to 2014, 17 studies comprising 185 patients who underwent 221 stenting procedures were reported. Mean prestent pressure gradient was 20.1 mmHg (95% CI 19.4–20.7 mmHg) with a mean poststent gradient of 4.4 mmHg (95% CI 3.5–5.2 mmHg). Complications occurred in 10 patients (5.4%; 95% CI 4.7–5.4%) but were major in only 3 (1.6%). At a mean clinical follow-up of 22 months, clinical improvement was noted in 130 of 166 patients with headaches (78.3%; 95% CI 75.8–80.8%), 84 of 89 patients with papilledema (94.4%; 95% CI 92.1–96.6%), and 64 of 74 patients with visual symptoms (86.5%; 95% CI 83.0–89.9%). In-stent stenosis was noted in six patients (3.4%; 95% CI 2.5–4.3%) and stent-adjacent stenosis occurred in 19 patients (11.4%; 95% CI 10.4–12.4), resulting in restenting in 10 patients. *Conclusion*. In IIH patients with venous sinus stenosis and a physiologic pressure gradient, venous stenting appears to be a safe and effective therapeutic option. Further studies are necessary to determine the long-term outcomes and the optimal management of medically refractory IIH.

## 1. Introduction 

Idiopathic intracranial hypertension (IIH) has long been associated with the hallmark clinical triad of headaches, papilledema, and visual loss in the absence of neurologic signs (with the exception of possible CN VI palsy), ventriculomegaly, or intracranial masses on CT or MRI [1]. While the incidence of IIH is relatively low among the general population at 1-2 per 100000 [1], it can be as high as 19–21 per 100000 in overweight, young adolescent to middle aged females [2]. To date, a variety of etiologies have been suggested to explain the pathophysiology behind IIH, including meningeal inflammation, metabolic disturbances (e.g., hyper- or hypoadrenalism and hypoparathyroidism), medication effects (e.g., excess vitamin A, corticosteroids, and tetracycline), and cerebral venous hypertension [3].

The first line treatment for IIH consists of weight loss and/or medical therapy including diuretics such as acetazolamide. When medical treatment fails, surgical options include cerebrospinal fluid (CSF) diversion via ventriculoperitoneal (VP) or lumboperitoneal (LP) shunting or optic nerve sheath fenestration. Recently, another etiology of cerebral venous hypertension has garnered increasing attention as a putative cause of IIH, cerebral venous dural sinus stenosis. In medically refractory IIH patients with a physiologic pressure gradient across venous stenosis, cerebral venous stenting has emerged as an alternative treatment option to traditional surgical approaches. While numerous groups have begun to use cerebral venous stenting for the treatment of IIH, the overall safety and efficacy remain unclear. The aim of this paper is to review the available literature on cerebral venous stenting for IIH with specific regard to patient complications, neurological outcomes, and radiographic results.

## 2. Methods

### 2.1. Inclusion Criteria

Studies for this systematic review were selected based on the following criteria: (1) the study must include at least one patient treated with cerebral venous sinus stenting for IIH, (2) the study must include posttreatment outcomes data, and (3) the language of the study must be in English. Studies pertaining only to alternative treatments for IIH were excluded.

### 2.2. Literature Search

A systematic review of the literature was performed using PubMed and the following search strategy: “Idiopathic Intracranial Hypertension” OR “Pseudotumor Cerebri” OR “Benign Intracranial Hypertension” OR “Venous Sinus Stenting.” A filter was used to only return articles written in English language reported after 1980.

### 2.3. Literature Review and Data Extraction

Information related to patient demographics, disease characteristics, treatment parameters, and poststent complications and outcomes were recorded from the studies that met the inclusion criteria. Whenever possible, we gathered specific demographic information from each study including body mass index (BMI), lumbar puncture opening pressure, mean prestent pressure gradient across the stenosis, and mean poststent pressure gradient across the stent.

Recorded data included number and percentage of treatment related complications and clinical outcomes, including improvement or deterioration of headache, vision, papilledema, and/or tinnitus. Major technical complications were defined as those requiring an intracranial intervention or resulting in a permanent neurological deficit. Follow-up imaging results were reviewed for in-stent stenosis and adjacent or out-of-stent stenosis, including retreatment rates.

### 2.4. Statistical Analysis

The statistical analysis in this review was performed using Stata version 8.0 (StataCorp LP, College Station, TX). Descriptive statistics were obtained for complications, neurological outcomes, and radiographic outcomes.

## 3. Results

### 3.1. Patient and IIH Characteristics

The literature review yielded 17 studies comprising 185 patients who underwent 221 venous stenting procedures. The mean patient age was 34.6 years, 161 patients (87%) were female, and the mean BMI was 33.4 Kg/m^2^. The most common presenting symptoms, in order of decreasing frequency, were headache in 89.7% (166/185 patients), papilledema in 63.6% (89/140 patients), visual decline in 60.7% (74/122 patients), and tinnitus in 50.9% (56/110 patients). The baseline patient and IIH characteristics are detailed in Table 1.

The mean opening pressure on lumbar puncture was 35.7 cmH_2_O (95% CI 34.8–36.2 cmH_2_O). The mean prestent pressure gradient was 20.1 mmHg (95% CI 19.4–20.7 mmHg) and the mean poststent pressure gradient was 4.4 mmHg (95% CI 3.5–5.2 mmHg). The overall mean change in gradient from prestent to poststent pressure gradient was 17.7 cmHg (95% CI 17.1–18.3 mmHg).

### 3.2. Periprocedural Complications

Complications were reported in 10 patients (5.4%; 95% CI 4.7–6.1%), including major complications in three patients (1.6%) and minor complications in seven patients (3.8%, Table 2). The major complications were two patients with subdural hemorrhages (SDHs) and one patient with both subarachnoid hemorrhage (SAH), ICH (intracerebral hemorrhage), and SDH. The minor complications were two patients with femoral artery pseudoaneurysms, two patients with transient hearing loss, one patient with a urinary tract infection (UTI), one patient with a syncopal episode, and one patient with a retroperitoneal hematoma. Four patients (2.1%; 95% CI 1.8–2.4%) required additional procedures as a result of complications, including craniotomy for SDH evacuation in two patients, external ventricular drain placement following SAH and SDH in one patient, and femoral artery stent placement for a pseudoaneurysm in one patient.

### 3.3. Neurological Outcomes

At a mean clinical follow-up of 22 months, patient improvement was noted for headaches in 130 of 166 patients (78.3%; 95% CI 75.8–80.8%), tinnitus in 52 of 56 patients (92.9%; 95% CI 88.7–97.1%), papilledema in 84 of 89 patients (94.4%; 95% CI 92.1–96.6%), and vision in 64 of 74 patients (86.5%; 95% CI 83.0–89.9%, Table 3).

### 3.4. Radiographic Outcomes

At a mean radiographic follow-up of 15.2 months, in-stent stenosis was noted in six patients (3.4%; 95% CI 2.5–4.3), but only one patient required retreatment (Table 4). Stent adjacent stenosis was more common, occurring in 19 patients (11.4%; 95% CI 10.4–12.4) and requiring treatment in 10 patients (6.0%; 95% CI 5.1–6.9).

## 4. Discussion 

The etiology of IIH has long been debated in the literature and currently remains elusive [21–23]. Intracranial venous hypertension, whether attributable to thrombosis, obstruction, or stenosis, is among the purported mechanisms underlying IIH. [24]. We briefly discuss the current treatments for IIH patients failing best medical therapy and weight loss, which have had varying degrees of success, before critically analyzing the role of cerebral venous sinus stenting.

### 4.1. Surgical Interventions for IIH

Surgical therapies are typically considered after medical therapy has failed and generally consist of CSF diversion (serial lumbar puncture, lumboperitoneal shunt, or ventriculoperitoneal shunt) or optic nerve sheath fenestration (ONSF). With regard to CSF diversion procedures, LP shunting is often preferred in IIH patients due to their characteristic silt-like ventricles which increase the difficulty of ventriculoperitoneal shunt placement. However, CSF diversion is fraught with hardware failure and repeated need for revisions along with infections. A recent review showed that, while LP and VP shunting are highly effective in mitigating IIH symptoms in the immediate postoperative period, both procedures have a fairly high failure rate. The revision rates for both forms of CSF diversion procedure were 60% for LP and 30% for VP shunts [25, 26].

ONSF is typically indicated in IIH patients with visual loss who endorse mild to no headaches. A small dural window created in the optic nerve sheath serves to drain CSF and relieve pressure on the optic disc, thereby helping to preserve vision. Another theory suggests that ONSF serves to elicit an inflammatory response that results in fibrosis of the optic nerve sheath, thereby preventing the transduction of intracranial pressure via the subarachnoid space to the optic disc. While ONSF often stabilizes vision function in the acute postprocedural period, it has been shown to have failure rates (defined as progressive vision loss after surgery) of 34% at 1 year and 45% at 3 years [15]. Thus, the current treatment options for IIH are limited by their lack of durability and relatively high long-term failure rates.

### 4.2. Role of Cerebral Venous Sinus Stenting in the Management of IIH

With the increasing recognition of cerebral venous stenosis as an etiology of IIH, dural venous sinus stenting has emerged as a potentially effective treatment. Recent publications have demonstrated promising clinical results with regard to headache and tinnitus resolution, papilledema reduction, and visual function improvement.

While the majority of the data focuses on headache improvement, more recent literatures have also focused on visual outcomes which may improve in a significant number of patients following stenting. However, many of the early studies simply state that treated patients' visual complaints improved without further quantification of pre- and postprocedural visual acuity or visual fields, and therefore it is hard to draw concrete conclusions from these studies [5–8, 13, 17]. More recent data have provided objective measures of ophthalmologic outcomes, including visual acuity and visual field testing [14, 18, 19]. As such, more rigorous ophthalmologic data will be needed in future studies to better substantiate the use of dural venous sinus stenting to improve vision in patients with IIH.

While venous stenting often obviates the need for CSF diversion, it is not without its own set of risks. Many of the reported complications arise from the angiography procedure rather than from stent placement. The most common complications were access related and include a retroperitoneal hematoma and 2 femoral pseudoaneurysms [15, 19, 20]. A more serious complication in the form of SDH and SAH was observed in 1 of 18 patients as reported by Kumpe et al. [16]. During stent placement of the right transverse sinus in this patient, there was stasis of flow in the right sigmoid sinus leading to a left SDH and SAH. The patient was managed successfully with an external ventricular drain. Similar complications were seen in a large series of 52 patients [14]. In this series, two patients had postprocedural SDHs. One patient developed a SDH after guidewire perforation of a dural sinus, while the other patient suffered a SDH along with SAH and intracerebral hemorrhage during emergent stent placement for fulminant IIH. Both patients underwent emergent craniotomies and made a full recovery. Although risks are inherent to any procedure, venous stenting for IIH remains a relatively safe procedure with numerous studies reporting no intraoperative complications [4–6, 11, 12, 18].

As with any stenting procedure, there exist complications inherently related to the stent, namely, in-stent stenosis. Two separate processes have been described for stent-related stenosis in the setting of IIH: in-stent stenosis and stent adjacent stenosis. Stent thrombosis may lead to in-stent stenosis or occlusion [27]. This, in general, would likely cause the return of the presenting symptoms. However, stent thrombosis may theoretically be disastrous if the thrombus occludes the drainage of the vein of Labbe. The increasing use of periprocedural dual antiplatelet therapy has led to a decrease in the incidence of in-stent stenosis [15, 19], although it has not been totally eliminated [20]. Stent adjacent stenosis is defined as a venous sinus stenosis which develops adjacent to the stent, often in the segment from the torcula to the stent, and is somewhat unique to the dural venous sinuses following stenting. This phenomenon has been described in 19 cases in this review [14–16], of which 10 underwent further stenting. However, some groups were elected to not treat asymptomatic stent adjacent stenosis. The phenomenon of out-of-stent stenosis in IIH raises the question as to whether there exists an inherent pressure from the brain parenchyma itself that serves to push on the venous sinus, giving them a stenosed appearance. Thus, venous sinus stenosis may be a result of idiopathic increased intracranial hypertension rather than a cause of it. Long-term radiographic outcomes and further delineation of the pathophysiology behind dural venous sinus stenosis are indicated in future studies.

Finally, it is important to consider that radiographic evidence of venous sinus stenosis alone is inadequate to justify stenting for IIH. There must also be physiologic evidence of a significant pressure gradient across the stenosis in order for stenting to be clinically efficacious. In our literature review, we found the mean prestent pressure gradient to be 20 mmHg. Further studies are necessary to determine the optimal gradient for stenting in IIH patients.

### 4.3. Study Limitations

This review is limited by the heterogeneity of the case series of which it is comprised. Specifically, there were no reporting standards for the baseline clinical and radiographic characteristics and for the posttreatment outcomes. Additionally, all studies were retrospective, and the number of patients per series was relatively small. Finally, the stent type and design varied across different series, thus limiting further the generalizability of our findings. Given these limitations, venous sinus stenting for patients with medically refractory IIH in whom a radiographic venous sinus stenosis and physiologic pressure gradient are both evident is a Class IIa Recommendation, Level of Evidence C.

## 5. Conclusions

Cerebral venous dural sinus stenting affords a favorable risk-to-benefit profile for appropriately selected IIH patients who are refractory to medical management and are demonstrated to have both a venous sinus stenosis and a physiologic pressure gradient. The available literature demonstrates that venous stenting is effective, but further long-term, prospective evaluation of this treatment approach is necessary. Specifically, additional studies that define ophthalmologic and radiographic baseline parameters and outcomes are requisite for defining the optimal patient population. Additionally, further work is necessary to determine the best therapeutic option for IIH patients.

## Figures and Tables

**Table 1:** Characteristics of patients and cases involved in stenting for IIH.

Author/year	Number of patients	Number of stents	Age	Female gender	BMI (Kg/m^2^)	CSF opening pressure (cm H_2_0)	Mean prestent pressure gradient (mmHG)	Mean poststent pressure gradient (mmHG)	Mean gradient change (mmHG)
Higgins et al. 2002 [4]	1	1	30	1/1	30.1	35	18	3	15
Owler et al. 2003 [5]	4	4	27 (17–38)	3/4	30 (23–48)	29 (22–35)^∗^	19 (12–25)	0.3 (0-1)	18.7
Higgins et al. 2003 [6]	12	14	33 (19–52)	12/12	36.9 (29–45)	33.7 (25–36)	18.9 (8–37)	11.3 (2–23)	7.6
Ogungbo et al. 2003 [7]	1	1	37	1/1	26.1	>40	25	NR	NR
Rajpal et al. 2005 [8]	1	1	15	0/1	26.9	37	25	NR	NR
Donnet et al. 2008 [9]	10	11	41 (28–60)	8/10	27.3 (22–37)	40.2 (29–59)	19.1 (12–34)	NR	NR
Paquet et al. 2008 [10]	1	1	60	1/1	NR	30	15	NR	NR
Arac et al. 2009 [11]	1	1	51	1/1	29	31	13	2	11
Bussière et al. 2010 [12]	10	13	34 (16–65)	10/10	35.9 (27–47)	NR	28.3 (11–50)	11.3 (2–23)	17
Zheng et al. 2010 [13]	1	1	34	1/1	26.1	40	22.5	6.5	16
Ahmed et al. 2011 [14]	52	60	34 (10–64)	47/52	>30 in 47	32.9 (25–73)^∧^	19.1 (4–41)	0.6 (0–14)	18.5
Albuquerque et al. 2011 [15]	15	30	32.3 (15–51)	12/15	NR	NR	NR	NR	NR
Kumpe et al. 2012 [16]	18	19	37.9 (16–62)	12/18	31.6 (22.6–38)	39.6 (25–55)	21.4 (4–39)	2.6 (0–7)	18.8
Teleb et al. 2012 [17]	1	1	22	1/1	28	48	26	0	26
Radvany et al. 2013 [18]	12	12	39 (21–55)	11/12	32.6 (27.3–45.7)	39.4 (29–55)	12.4 (5–28)	1.3 (0–4)	11.1
Fields et al. 2013 [19]	15	15	34 (20–56)	15/15	39 (30–73)	NR	24 (13–40)	4 (0–9)	20
Ducruet et al. 2014 [20]	30	36	33 (14–52)	25/30	NR	NR	NR	NR	21.4 (10–56)
Characteristics	Number of patients	Number of stents	Mean age (95% CI)	Female gender (%; 95% CI)	BMI (95% CI)	CSF opening pressure (95% CI)	Mean prestent pressure gradient (95% CI)	Mean poststent pressure gradient (95% CI)	Mean gradient (95% CI)
Summary	185	221	34.6 (34.0–35.1)	161 (87.0; 0.86–0.89)	33.4 (32.6–34.3)	35.7 (34.8–36.2)	20.1 (19.4–20.7)	4.4 (3.5–5.2)	17.7 (17.1–18.3)

^∗^Not reported in 1 patient.

^∧^Not reported in 11 patients.

**Table 2:** Complications following stenting for IIH.

Author/year	Number of patients	Number of stentings	Complications	Complication rate	Complications requiring additional procedure
Higgins et al. 2002 [4]	1	1	0	0%	N/A
Owler et al. 2003 [5]	4	4	0	0%	N/A
Higgins et al. 2003 [6]	12	14	0	0%	N/A
Ogungbo et al. 2003 [7]	1	1	0	0%	N/A
Rajpal et al. 2005 [8]	1	1	0	0%	N/A
Donnet et al. 2008 [9]	10	11	0	0%	N/A
Paquet et al. 2008 [10]	1	1	0	0%	N/A
Arac et al. 2009 [11]	1	1	0	0%	N/A
Bussière et al. 2010 [12]	10	13	0	0%	N/A
Zheng et al. 2010 [13]	1	1	0	0%	N/A
Ahmed et al. 2011 [14]	52	60	2 major (SDH); 2 minor (transient hearing loss)	7.7%	2 (1 SDH, 1 SDH/ICH/SAH both requiring emergent craniotomy)
Albuquerque et al. 2011 [15]	15	30	1 minor RPH not requiring transfusion	3.3%	0
Kumpe et al. 2012 [16]	18	19	1 major (SAH/SDH); 2 minor (UTI and syncope)	16.7%	1 (SAH/SDH hematoma requiring EVD)
Teleb et al. 2012 [17]	1	1	0	0%	N/A
Radvany et al. 2013 [18]	12	12	0	0%	N/A
Fields et al. 2013 [19]	15	15	1 minor (femoral pseudoaneurysm)	6.7%	0 (femoral pseudoaneurysm resolved compression)
Ducruet et al. 2014 [20]	30	36	1 minor (femoral pseudoaneurysm	2.8	1 (femoral pseudoaneurysm requiring femoral artery stent)
Characteristics	Number of patients	Number of stents	Complications	Complication rate % (95% CI)	Complications requiring additional procedure (%; 95% CI)
Summary	185	221	10	5.4% (4.7–6.1)	4 (2.1%; 1.8–2.4%)

SAH: subarachnoid hemorrhage.

SDH: subdural hemorrhage.

ICH: intracerebral hemorrhage.

RPH: retroperitoneal hematoma.

**Table 3:** Neurologic outcomes following stenting for IIH.

Author/year	Number of patients	Number of stents	Improved headache	Improved tinnitus	Improved papilledema	Improved vision	Follow-up
Higgins et al. 2002 [4]	1	1	1/1	NA	1/1	1/1	12
Owler et al. 2003 [5]	4	4	3/4	1/1	4/4	4/4	9.8 (5–12)
Higgins et al. 2003 [6]	12	14	7/12	NR	5/8	7/12	14.2 (2–26)
Ogungbo et al. 2003 [7]	1	1	1/1	NA	1/1	1/1	6
Rajpal et al. 2005 [8]	1	1	1/1	NA	1/1	1/1	6
Donnet et al. 2008 [9]	10	11	8/10	9/9	10/10	9/10	17.2 (6–36)
Paquet et al. 2008 [10]	1	1	1/1	NA	1/1	1/1	NR
Arac et al. 2009 [11]	1	1	1/1	0/1	NA	NA	2
Bussière et al. 2010 [12]	10	13	10/10	3/3	9/9	7/8	20.1 (4–60)
Zheng et al. 2010 [13]	1	1	1/1	NA	1/1	1/1	3
Ahmed et al. 2011 [14]	52	60	40/43	17/17	9/9	19/19	24 (2–108)
Albuquerque et al. 2011 [15]	15	30	12/15 (1: worse, 2: no change)	NR	NR	NR	20 (2–40)
Kumpe et al. 2012 [16]	18	19	10/12 (2: no change)	NR	15/16	NR	43.7 (11–136)
Teleb et al. 2012 [17]	1	1	1/1	NA	1/1	1/1	6
Radvany et al. 2013 [18]	12	12	5/12 (5: no change)	11/11	11/12	10/12	16 (9–36)
Fields et al. 2013 [19]	15	15	10/15 (1: no change, 2: worse, 2: different)	11/14	15/15	2/3	14 (1–49)
Characteristics	Number of patients	Number of stents	Improved headache (%; 95% CI)	Improved tinnitus (%; 95% CI)	Improved papilledema (%; 95% CI)	Improved vision (%; 95% CI)	Mean follow-up (95% CI)
Ducruet et al. 2014 [20]	30	36	18/26 (8: no change)	NR	NR	NR	23 (0–58)
Summary	185	221	130 (78.3; 75.8–80.8)	52 (92.9; 88.7–97.1)	84/89 (94.4; 92.1–96.6)	64/74 (86.5; 83.0–89.9)	22.0 (20.7–23.2)

**Table 4:** Radiographic outcomes following stenting for IIH.

Author/year	Number of patients	Number of stentings	Average radiographic follow-up (months)	Number of in-stent stenoses	Subsequent treatment	Number of out-of-stent stenoses	Subsequent treatment
Higgins et al. 2002 [4]	1	1	1	0	0	0	0
Owler et al. 2003 [5]	9	4	3	0	0	1	0
Higgins et al. 2003 [6]	12	14	NR	2	Thrombolytic therapy	NR	NR
Ogungbo et al. 2003 [7]	1	1	12	0	0	0	0
Rajpal et al. 2005 [8]	1	1	6	0	0	0	0
Donnet et al. 2008 [9]	10	11	6	0	0	0	0
Paquet et al. 2008 [10]	1	1	NR	NR	NR	NR	NR
Arac et al. 2009 [11]	1	1	2	0	0	0	0
Bussière et al. 2010 [12]	10	13	NR	NR	NR	NR	NR
Zheng et al. 2010 [13]	1	1	NR	NR	NR	NR	NR
Ahmed et al. 2011 [14]	52	60	NR	0	0	6	Resenting in 6 patients
Albuquerque et al. 2011 [15]	15	30	12.5	0	0	1	Restenting
Kumpe et al. 2012 [16]	18	19	25.3	0	0	4	Restenting in 1 patient
Teleb et al. 2012 [17]	1	1	12	0	0	0	0
Radvany et al. 2013 [18]	12	12	NA	0	0	2	Restenting in 2 patients and 1 patient requiring VPS
Characteristics	Number of patients	Number of stentings	Average radiographic follow-up (95% CI)	Number of in-stent stenoses (%; 95% CI)	Subsequent treatment (%)	Number of out-of-stent stenoses	Subsequent treatment
Fields et al. 2013 [19]	15	15	9	0	0	0	0
Ducruet et al. 2014 [20]	30	36	22	4	0	5	0
Summary	185	221	15.2 (13.6–16.8)^1^	6 (3.4; 2.5–4.3)^2^	1 (0.6)^∧^	19 (11.4; 10.4–12.4)^3^	10 (6.0; 5.1–6.9)^3^

^1^Reported in 11 studies (*N* = 102).

^2^Reported in 14 studies (*N* = 178).

^3^Reported in 13 studies (*N* = 166).

^∧^Refers to 1 patient who received thrombolytic therapy. Not patients received restenting.
